# Organelle acidification: an ancient cellular leak detector

**DOI:** 10.1186/s12915-017-0395-1

**Published:** 2017-06-26

**Authors:** Mukund Thattai

**Affiliations:** Simons Centre for the Study of Living Machines, National Centre for Biological Sciences, Tata Institute of Fundamental Research, GKVK Campus, Bellary Road, Bangalore, 560065 India

## Abstract

Intracellular membrane-bounded organelles of eukaryotic cells transiently contact the extracellular environment during endocytosis and secretion. Such contacts must be precisely timed to prevent leakage of cargo. I argue that early eukaryotes evolved organelle acidification as a way to detect and prevent leakage.

## The evolution of endocytic compartments in eukaryotic cells

Eukaryotes differ from prokaryotes along many parameters: size and shape, membrane organization, chromosomal structure, and reproductive modes. But a quintessential eukaryotic trait, one which possibly enabled early eukaryotes to colonize new and diverse niches, is the capacity for intracellular digestion. All living cells face the challenge of extracting nutrients from environments in which food molecules are too large to import across the plasma membrane. Cells in all three domains of life solve this problem the same way: they use hydrolase enzymes to break down the food into more manageable chunks, which are then imported. Bacteria and archaea extract nutrients by co-translationally or post-translationally secreting hydrolases directly into the external environment. This strategy has two negative consequences [1]. First, it raises the possibility of cheating: enzyme production and secretion impose a private cost, but the resulting metabolic products are public goods which can benefit any nearby cell. Second, enzymes secreted into the extracellular milieu cannot be re-used at a later time. These issues can be partly mitigated: cells could grow to high density to increase nutrient capture; secreted proteins could be retained in the periplasmic space (an option not available to single-membrane-bounded archaea) or they could be membrane-anchored. Nevertheless, a significant fraction of prokaryotic secreted hydrolases and nutrient breakdown products diffuse away, never to be recovered.

In this context, the eukaryotic innovation was to “sip” their nutrient-rich surroundings into intracellular membrane-bounded compartments collectively called endosomes. Modern eukaryotes take up specific cargo molecules using hundred-nanometre-scale coated vesicles, and internalize large prey particles or the general fluid milieu in micron-scale carriers via phagocytosis or pinocytosis [2]. Lysosomes and secretory vesicles fuse with maturing endosomes, delivering the hydrolase enzymes which break down the available nutrients. The breakdown products cross into the cytoplasm via transporters and pumps, and the hydrolases are used for a new round of digestion. Just as for prokaryotic extracellular digestion, eukaryotic intracellular digestion requires the transport of enzymes across a membrane. Eukaryotes co-translationally transport proteins into the lumen of the endoplasmic reticulum, and then deliver them via secretory vesicles to endosomes and lysosomes. Crucially, though the organellar lumen is topologically equivalent to the extracellular space, it is never directly connected to it. In this way, eukaryotes avoid leaking enzymes and nutrients into the environment.

## Vesicle acidification allows leak detection

**Fig. 1.**
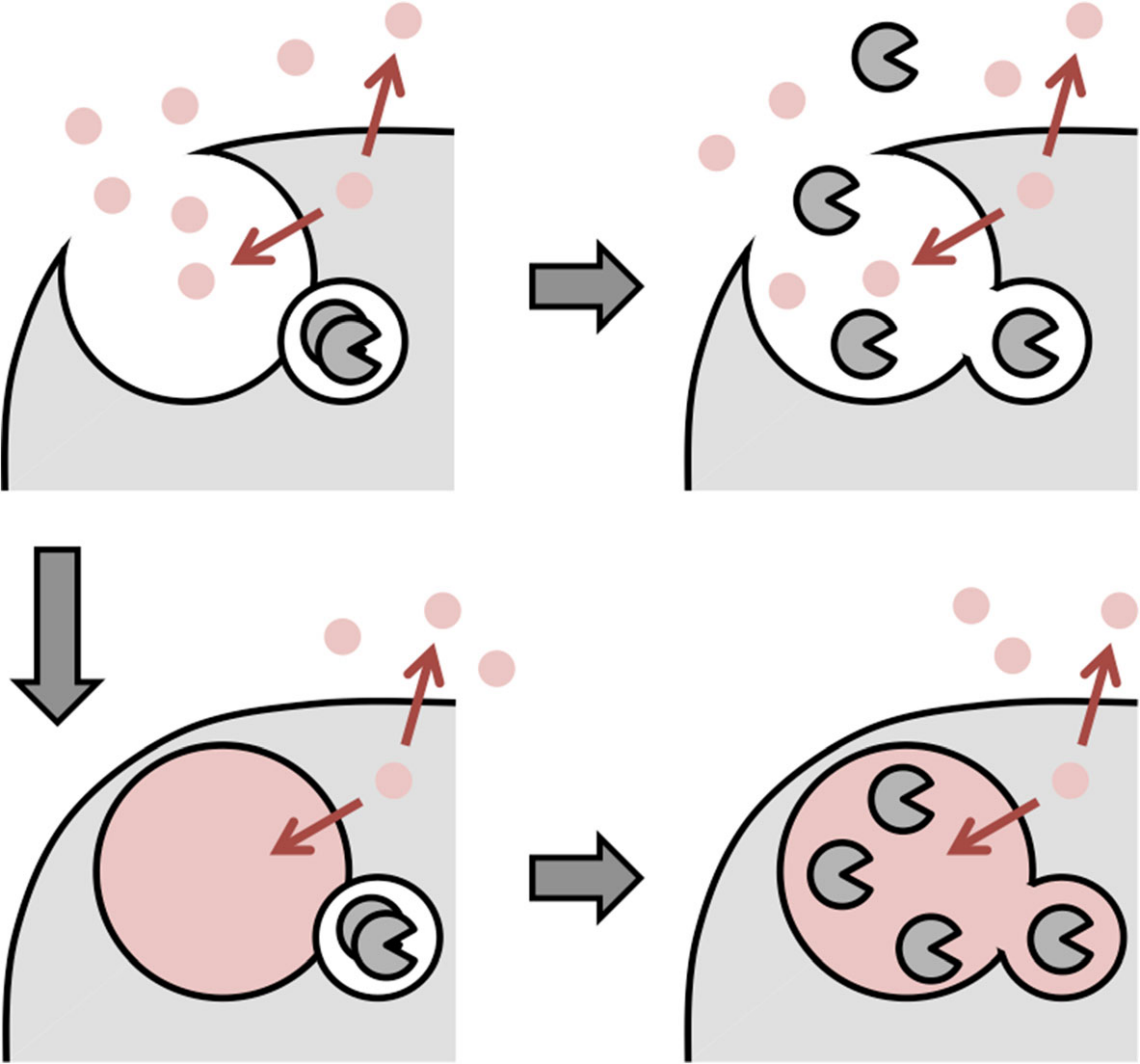
The principle of leak detection. A dye, shown in *pink*, is constantly pumped across the plasma membrane. *Top left*: the volume enclosed by a membrane invagination is connected to the extracellular environment. *Top right*: enzymes delivered before a seal develops will leak away. *Bottom left*: the membrane invagination is sealed off from the environment, causing the dye to accumulate. *Bottom right*: enzymes delivered once the dye accumulates will not leak away. This principle applies both to endocytic as well as secretory organelles that are transiently connected to the extracellular environment. Eukaryotes universally use protons as the leak-detection dye, pumped by V-ATPases

But this scenario raises an engineering problem. How do nanometre-scale vesicle fusion regulators know that micron-scale endocytic structures have been fully sealed from the environment? Premature delivery of secretory vesicles before a seal is established would result in a costly enzyme leak (Fig. 1, top). The simplest way to build a leak detector, as in many real-world engineering applications, is to constantly pump a dye into the developing endocytic structure. As long as there is no seal, the dye will leak into the environment; once a seal is established the dye will accumulate, signalling that it is safe to deliver enzymes (Fig. 1, bottom). The ideal dye should be inexpensive, abundant, rapidly diffusing, and easily sensed. Essentially the only dye that satisfies these four criteria is the proton. Protons arise from the equilibrium dissociation of water; pH 7 corresponds to 100 protons per cubic micron, pH 5 to 10,000 protons per cubic micron. Though protons under physiological conditions exist as bulky hydronium ions (H_3_O^+^), they diffuse rapidly by hopping along hydrogen bonds [3]: the diffusion coefficient of protons is ~ 10 μm^2^/ms, several times that of water (~2.5 μm^2^/ms) and Na^+^ (~ 1.5 μm^2^/ms). Finally, almost all proteins are pH-sensitive via amino acid protonation and de-protonation.

**Fig. 2.**
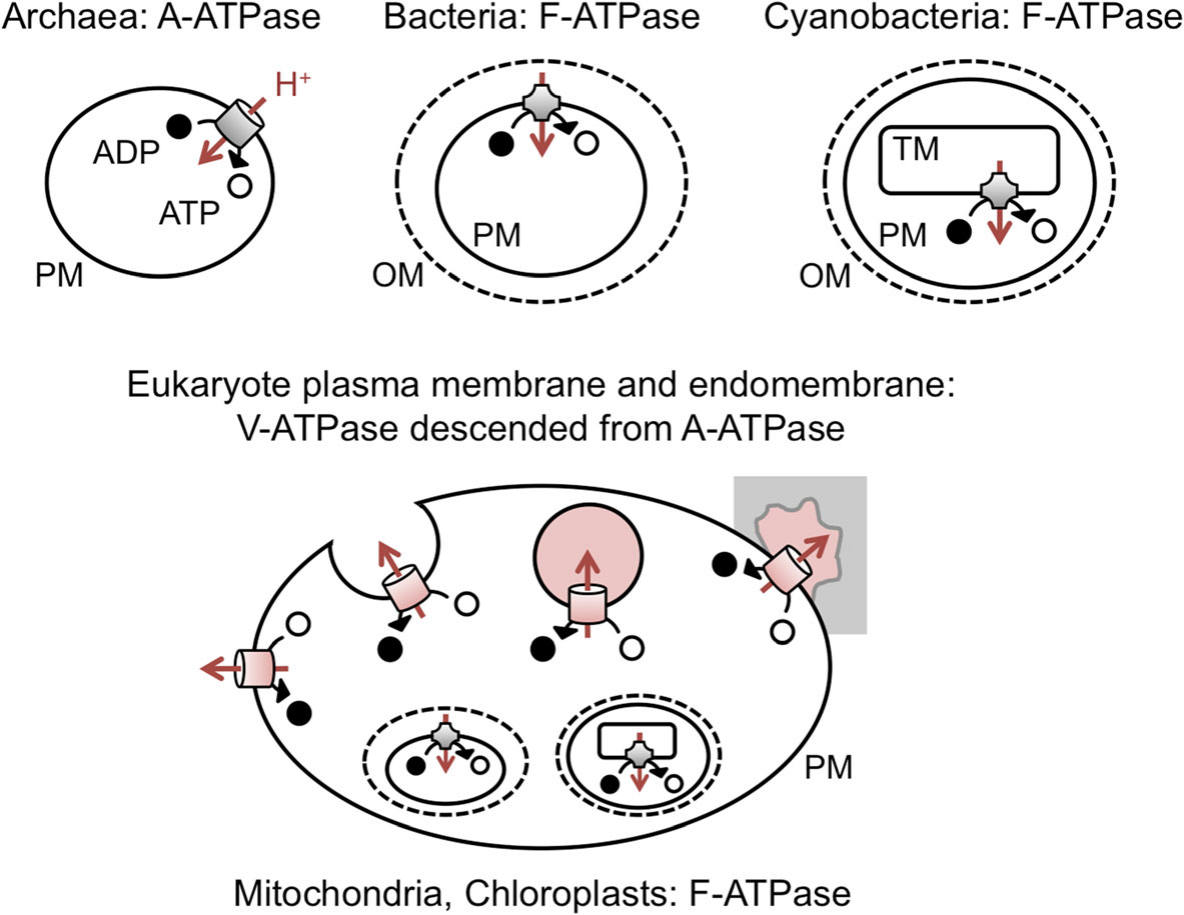
Distribution and orientation of rotary-motor proton pumps across the three domains of life. Archaeal A-ATPases and bacterial (including cyanobacterial) F-ATPases use the flow of protons into the cytoplasm to convert ADP to ATP. This is balanced by an energy-dependent outward flow driven by the electron transport chain. Eukaryotic mitochondria and chloroplasts are endosymbiotic descendants of alpha-proteobacteria and cyanobacteria, and continue to use F-ATPases like their free-living ancestors. Eukaryotic V-ATPases are derived from archaeal ATPases but run in the opposite direction: they hydrolyse ATP to drive protons into the extracellular environment, the organellar lumen, or sealed extracellular pockets. The flow of protons out of the cytoplasm is balanced by an inward flow via various symporters and antiporters. The flow of protons into endosomes and extracellular pockets is unbalanced, leading to their acidification (*pink*). *OM* outer membrane, *PM* plasma membrane, *TM* thylakoid membrane

Indeed, protons play a central role in eukaryotic membrane traffic [4]. The transmembrane vacuolar ATPase (V-ATPase) protein uses the free energy of ATP hydrolysis to constantly pump protons from the cytoplasm across the plasma membrane into the extracellular environment (Fig. 2). V-ATPases also drive protons across the invaginating membranes of nascent endocytic structures. Once these structures pinch off from the plasma membrane, they are rapidly acidified by the unbalanced flow of protons into the newly formed lumen. A single V-ATPase under physiological conditions can pump a hundred protons per second, so a hundred V-ATPases acting together will bring a micron-scale endosome to pH 5 in about a second [5]. This acidification acts as a trigger for vesicle fusion and enzyme delivery.

The V-ATPase system satisfies all the requirements of leak detection. But do cells actually use this capability? After all, leak detection by protons is always coupled to acidification; perhaps acidification itself is the primary function. It is commonly believed that endosomal and lysosomal acidification are essential for the digestive activity of lysosomal enzymes, which are called acid hydrolases because of their acidic pH optima. I suggest instead that endosomal acidification first arose for leak detection, and that lysosomal hydrolases subsequently adapted to function under acidic conditions. Many lines of evidence support this idea. The first is the precise timing of events. If digestion were the only concern, endosomal acidification and enzyme delivery could proceed simultaneously, yet they do not. Instead, secretory vesicles and lysosomes fuse only to pre-acidified endosomes, and some lysosomal enzymes even remain membrane anchored via the mannose-6-phosphate receptor until released in an acidic environment [4], as required for leak detection. The second is that it is unlikely that acidity is essential for hydrolase activity. Enzymes across the tree of life have evolved to function under various pH ranges, and often a few amino acid substitutions are sufficient to convert a protein’s optimal activity range from acidic to neutral pH [6]. Bacterial hydrolases function as efficiently under neutral conditions as lysosomal hydrolases do under acidic conditions. Moreover, eukaryotic lysosomal hydrolases can operate at neutral pH. Yeast *vma* mutants with V-ATPase defects are viable in pH 5 media: a condition in which endosomes are acidic but lysosomes are neutral [7]. Yet these mutants fail to grow in neutral media because their lysosomes cannot fuse with unacidified endosomes: a hydrolase delivery defect rather than a hydrolase activity defect. Taken together, these observations lend weight to the proposition that the pH-triggered delivery of hydrolase enzymes, thereby avoiding leaks, is a key function of endosomal acidification.

V-ATPases are also active in secretory contexts [4] in which there is no obvious benefit of acidification per se, but the benefits of leak detection are compelling. There are many examples of secretory organelles being assembled ahead of time, later releasing their cargo in a precisely timed pulse. Synaptic vesicles rapidly fuse to the plasma membrane upon stimulation of a neurone, releasing a pulse of neurotransmitters, and are then slowly retrieved [8]. It is essential that neurotransmitters are only delivered to the intracellular vesicle pool, and not to vesicles transiently fused to the plasma membrane. Indeed, neurotransmitter transport into synaptic vesicles is contingent on their acidification by V-ATPases; vesicles fused to the plasma membrane rapidly de-acidify, preventing continuous neurotransmitter leak [8]. Similarly, but at a very different scale, the biogenesis of the micron-scale multi-vesicular body in which exosomes are stored prior to secretion depends on an acidified lumen via V-ATPase activity [4]. A slightly different example is extracellular lysosomal activity. Macrophages in contact with lipoprotein aggregates or osteoclasts in contact with bone form digestive extracellular pockets [4]. Only once these structures are tightly sealed do they acidify via plasma membrane V-ATPase activity, triggering the export of lysosomal enzymes (Fig. 2, bottom).

Leak detection requires the ability to both drive as well as sense acidification. Interestingly, components of the V-ATPases themselves might play a role in pH sensing [4]. The co-evolution of pH-generating and pH-sensing capabilities of V-ATPases could explain why these proteins are the main drivers of acidification in diverse cellular contexts where leak detection is expected to be important. The leak detection hypothesis provides the most parsimonious account of an otherwise heterogeneous distribution of acidified organelles across the eukaryotic cell: intracellular compartments that transiently come into contact with the extracellular environment are acidified by V-ATPases (endosomes, lysosomes, synaptic vesicles, multi-vesicular bodies), whereas those that are purely intracellular are near-neutral (endoplasmic reticulum, Golgi apparatus, mitochondrial matrix, chloroplast stroma). Once an acidification mechanism is available, it can of course be exapted for functions other than leak detection. For example: acidic environments enhance or inhibit a variety of chemical reactions; intracellular pH and potential gradients can power endomembrane symporters and antiporters; and the sustained acidification of endosomes converts a simple yes/no question ("has the compartment internalised?") into a useful timing mechanism ("how long since the compartment internalised?").

## Phylogenetics suggests how acidification evolved

We can use biophysical and molecular phylogenetic data to probe the evolution of V-ATPases, shedding light on how acidification first evolved. All living cells use the same ancient proton-dependent mechanism to generate ATP, in order to supplement inefficient glycolysis. Cells first use energy from photosynthesis, aerobic respiration, or other chemical sources to pump protons across a membrane, out of the cytoplasm. When these protons flow back down the gradient into the cytoplasm, they propel a rotary motor, driving ATP synthesis [5]. In bacteria, mitochondria, and chloroplasts, this is done by the F-ATPase rotary motors; in archaea this is done by A-ATPase rotary motors [9] (Fig. 2). Eukaryotes are thought to have arisen via a symbiosis between bacterial and archaeal ancestors about two billion years ago, the former evolving into mitochondria and the latter providing the plasma membrane (for one reconstruction of how this might have happened see [10]). A subsequent symbiotic partnership with photosynthetic cyanobacteria gave rise to chloroplasts. Molecular phylogenetics strongly supports this scenario: bacterial F-ATPases are localized to the mitochondrial and chloroplast inner membrane, while V-ATPases derived from archaeal ATPases are localized to the eukaryotic plasma membrane [9] (Fig. 2). Present-day bacterial F-ATPases and archaeal A-ATPases can operate in two directions: using inward proton flow to propel the rotary ATP synthase motor in photosynthetic or aerobic conditions, or using ATP hydrolysis to pump protons outward in anaerobic conditions. However, these bidirectional proteins cannot pump protons against large pH gradients [5, 9]. Strikingly, all eukaryotic V-ATPases have lost the ability to operate as motors: they work exclusively as pumps, due to a reduced H+/ATP coupling ratio [5, 9]. In the process, V-ATPases gained an ability their A-ATPase ancestors lacked: the capacity to generate and maintain large pH gradients.

These data support the well established idea that organelle acidification was a key early eukaryotic adaptation. But we should still ask what drove this innovation billions of years ago. The answer is necessarily speculative. Here I have argued, based on cell-biological and phylogenetic data, that leak detection must be considered alongside any other hypotheses. When we think of cells as engineers would, acidification seems an elegant way to solve a problem the first eukaryotes would have faced: how to detect whether intracellular compartments were tightly sealed.
